# Comparative Evaluation of Resting-State fMRI Representations for Schizophrenia Classification Using Deep Learning

**DOI:** 10.31083/AP53200

**Published:** 2026-10-16

**Authors:** Şerife Gengeç Benli, Semra İçer, Turgay Batbat, Zeynep Ak

**Affiliations:** ^1^Department of Biomedical Engineering, Engineering Faculty, Erciyes University, 38030 Kayseri, Turkiye; ^2^Department of Biomedical Engineering, Graduate School of Natural and Applied Sciences, Erciyes University, 38039 Kayseri, Turkiye

**Keywords:** schizophrenia, fMRI, data representation, CNN, ALFF

## Abstract

**Background:**

Schizophrenia is a complex and heterogeneous psychiatric disorder characterized by widespread impairments in cognitive, emotional, and perceptual processes. The increasing demand for objective, biologically grounded biomarkers in clinical diagnostics has catalyzed significant interest in functional Magnetic Resonance Imaging (fMRI) integrated with deep learning-based methodologies. However, the prevailing literature relies on pre-derived features such as functional connectivity matrices, independent component analysis outputs, and time-series representations. These approaches often introduce additional computational costs, methodological complexity, and sensitivity to parameter selection, thereby limiting scalability.

**Methods:**

To address these limitations, this study proposes a simplified end-to-end deep learning framework that does not require derived features and instead learns directly from preprocessed fMRI (pre-fMRI) data. In addition, an Amplitude of Low-Frequency Fluctuation (ALFF)-based dimensionality reduction strategy was employed, enabling direct classification from resting-state fMRI data without requiring functional connectivity or correlation matrix computation. Resting-state fMRI data from publicly available datasets, including participants aged 18–62 years (individuals with schizophrenia: 78; healthy controls: 158), were modelled using multiple representations, including three-dimensional (3D) blood oxygen level-dependent (BOLD) volumes, ALFF maps, mean- and standard deviation-based statistical summaries, and four-dimensional (4D) spatiotemporal structures. These representations were evaluated using Convolutional Neural Network (CNN)-based approaches with five-fold cross-validation.

**Results:**

The findings clearly demonstrate that data representation, rather than model architecture, serves as the primary determinant of classification performance. The highest performance was achieved using ALFF-based 3D representations, with an accuracy of 80.10%. In contrast, 4D approaches that jointly model spatial and temporal information did not yield a significant performance improvement despite increased architectural complexity and computational cost. This suggests that biologically meaningful and information-rich signal representations play a more critical role than model depth or temporal modelling strategies.

**Conclusions:**

The proposed approach demonstrates that effective classification can be achieved without relying on complex derived features such as functional connectivity, offering a simpler, more computationally efficient, and generalizable framework. The findings challenge the prevailing trend toward increasing architectural complexity in the literature and emphasize the critical importance of data representation. This study provides valuable insights for developing more scalable, interpretable, and clinically applicable artificial intelligence models in fMRI-based studies.

## Main Points

1. This study presents a comparative analysis of classification performance across multiple fMRI data representations, including the one-sample volume, ALFF-based mean volume, mean volume and standard deviation-based volume configurations.

2. Performance variations depending on data representation are investigated using DarkNet-53 and ResNet-50 models.

3. The finding that the ALFF-based configuration yields the highest accuracy highlights the discriminative power of this approach. It supports the critical role of low-frequency BOLD signal fluctuations in the pathophysiology of schizophrenia.

4. In addition to 3D approaches, 4D spatiotemporal modelling is also evaluated using a DarkNet-19 + LSTM architecture.

## 1. Introduction

Schizophrenia is a heterogeneous and chronic psychiatric disorder characterized by widespread impairments in cognitive, emotional, and perceptual processes [1,2,3,4]. The diagnostic process largely relies on clinical observation and symptom-based assessments, and there is an increasing need for objective and biologically grounded biomarkers [5,6]. In this context, functional magnetic resonance imaging (fMRI) has become an important tool for elucidating the neurobiological underpinnings of schizophrenia, particularly in task-based and resting-state paradigms [7,8,9]. In recent years, advances in deep learning and machine learning have enabled highly accurate automatic classification from fMRI data, leading to widespread development of computer-aided diagnostic systems in the literature.

In task-based fMRI studies, activation maps obtained in response to specific cognitive or sensory stimuli have been used. For instance, Oh et al. [10] analysed three-dimensional (3D) activation maps derived from a visual-auditory task paradigm using a 3D Convolutional Autoencoder (CAE)-Convolutional Neural Network (CNN) architecture and reported an accuracy exceeding 84%. Similarly, Dakka et al. [11] classified four-dimensional (4D) fMRI data acquired during an auditory oddball task from the FBIRN dataset using Long Short-Term Memory (LSTM) and R-CNN architectures, achieving higher performance compared to traditional Support Vector Machine (SVM) methods. These studies demonstrate that task-related activation patterns can be effectively discriminated using deep learning approaches; however, dependency on task design is considered a limiting factor for clinical applicability.

In resting-state fMRI (rs-fMRI) studies, functional connectivity (FC) matrices [12], independent component analysis (ICA)-based time series [13], and connectivity patterns derived from these components are commonly used as input to deep learning models. In addition, various voxel-wise measures such as the amplitude of low-frequency fluctuations (ALFF), fractional ALFF (fALFF), regional homogeneity (ReHo), and voxel-mirrored homotopic connectivity (VMHC) [14] are also employed as input features. Lei et al. [15] demonstrated that a Graph Convolutional Network (GCN) model achieved an accuracy of 85.8% on FC matrices in a large multi-site dataset, outperforming SVM. Similarly, Zhao et al. [16] reported an accuracy above 82% using a Generative Adversarial Network (GAN) on ICA-based connectivity matrices obtained from seven different sites. Furthermore, Yan et al. [17] achieved an accuracy of 83.2% using multi-scale recurrent neural networks combined with a multi-site pooling strategy. These findings suggest that functional connectivity patterns, in particular, may serve as strong and discriminative biomarkers for schizophrenia.

Although rs-fMRI approaches that do not rely on functional connectivity measures are relatively limited in the literature, slice-wise 2D CNN architectures and transfer learning strategies have reported accuracy rates of up to 99% [18,19]. In these studies, data-driven optimization steps, such as reducing 4D fMRI data to 2D slices and selecting discriminative slices and time points, significantly enhance performance. However, such high performance may be highly dependent on data selection and should therefore be carefully evaluated for model generalizability.

In addition, structural MRI (sMRI) studies play a critical role in revealing morphological alterations in the brain associated with schizophrenia [20,21,22]. Findings such as gray matter volume reduction, ventricular enlargement, and subcortical structural differences have been reported in the literature [23,24,25]. Tyagi et al. [25] achieved up to 99.2% accuracy using CNN-based deep learning along with k-NN, SVM, and Random Forest (RF) classifiers on 2D slices derived from Center for Biomedical Research Excellence (COBRE) and OpenfMRI datasets. Zhang et al. [24] analysed whole-brain 3D T1-weighted images using a 3D VGG architecture and reported accuracy above 90%, with findings in subcortical and ventricular regions consistent with previously reported anatomical alterations. Furthermore, Vyškovský et al. [23] employed stacked autoencoders and 3D CNN approaches on a male-only dataset to analyse gray matter segmentation and local volumetric changes, achieving high classification performance and regional discriminability. Additionally, Gengeç Benli and Andaç [26] extracted Gray-Level Co-Occurrence Matrix (GLCM) features from bilateral amygdala, caudate, pallidum, putamen, and thalamus regions and compared various machine learning methods, reporting 94% accuracy with LDA. These findings indicate that sMRI-based deep learning approaches offer significant advantages for schizophrenia diagnosis, with high accuracy and greater interpretability of regional brain contributions.

In most studies in the literature, pre-derived features such as connectivity matrices, graph-based brain topography, ICA components, correlation-based features, or activation maps are used as input to deep learning models. This introduces additional computational burden, methodological complexity, and variability due to parameter selection. Moreover, differences in atlas selection, correlation metrics, thresholding strategies, functional connectivity analysis tools, and feature selection methods complicate the comparability of results across studies. In contrast, the present study aims to use preprocessed fMRI (pre-fMRI) image representations directly as input to deep learning architectures, enabling a systematic comparison of minimally processed images and biologically derived representations without relying on functional connectivity analysis. CNN-based architectures, including DarkNet-53, ResNet-50, DarkNet-19, and LSTM, were applied to pre-fMRI data from the COBRE and UCLA datasets.

Overall, rather than proposing a new deep learning architecture, this study provides an alternative representation-focused perspective by systematically investigating how different representations of preprocessed fMRI images influence schizophrenia classification performance. This approach offers methodological simplicity and computational efficiency while demonstrating that biologically meaningful image representations may contribute more substantially to classification performance than increasing architectural complexity.

## 2. Materials and Methods

In this study, a deep learning-based analytical framework was developed to classify individuals with schizophrenia and healthy control groups using functional magnetic resonance imaging (fMRI) data. During the analysis, pre-fMRI data were transformed into 3D and 4D representations that include temporal information, which were used as inputs to the models. In the 3D approach, volumetric data were processed directly while preserving their spatial integrity. In the 4D representation, temporal dynamics were captured by analysing different time windows, and hybrid architectures combining CNNs and recurrent neural networks (RNNs) were employed to model temporal dependencies. ResNet-50 and DarkNet-53 were selected as deep learning models for automatic feature extraction and classification. In addition, LSTM layers were used to incorporate temporal dependencies into the model, leveraging data that captures temporal information. To compare the performance of different data representations and model architectures, five-fold cross-validation was applied in all experiments, thereby improving model generalizability and reducing the risk of overfitting. The results were evaluated using accuracy, F1-score, and area under the curve (AUC); subsequently, the classification performance of different approaches was systematically analysed. The preprocessing steps, the dimensional characteristics of the imaging data, and the analytical procedures used in this study are illustrated in Fig. 1.

**Fig. 1.**
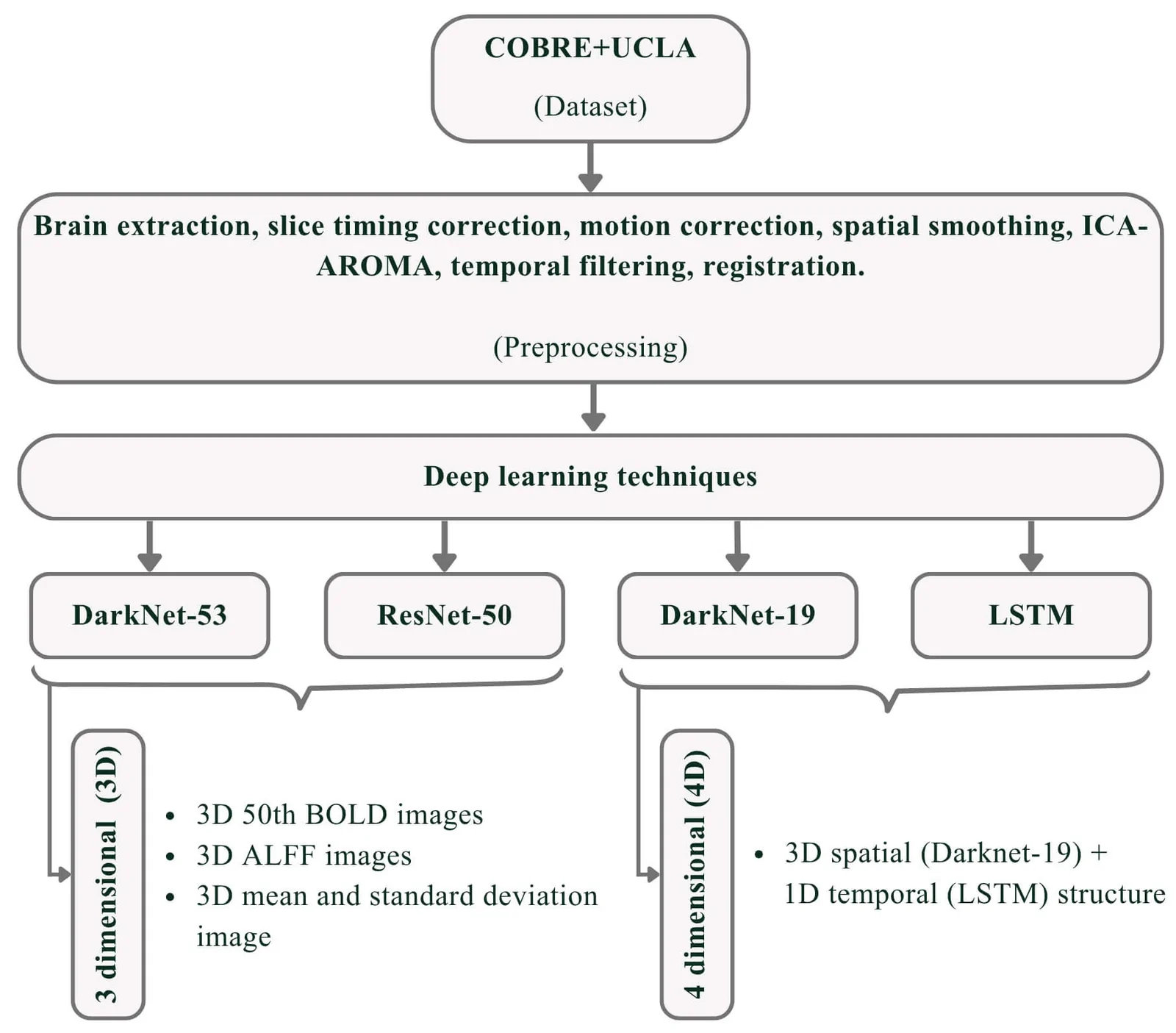
**Overview of the study design and analysis workflow**. The diagram summarizes the overall pipeline, including dataset selection, preprocessing, deep learning model design for data representation, and evaluation of different fMRI representations. Abbreviations: ICA-AROMA, Independent Component Analysis–Automatic Removal of Motion Artifacts; ALFF, Amplitude of Low-Frequency Fluctuation; LSTM, Long Short-Term Memory.

### 2.1 Dataset

The UCLA dataset consists of participants aged between 21 and 50 years (mean: 33.23; median: 31.0), including healthy controls (n = 130; 155 males and 117 females) as well as individuals diagnosed with adult attention deficit hyperactivity disorder (ADHD) (n = 43), bipolar disorder (n = 49), and schizophrenia (n = 50) [27]. Individuals with diagnoses from at least two different patient groups were excluded. Additional exclusion criteria included left-handedness, pregnancy, a history of head trauma associated with loss of consciousness, and other contraindications for MRI scanning. MR images were acquired using a 3T Siemens Trio scanner at the Ahmanson-Lovelace Brain Mapping Center (Siemens version syngo MR B15) and the Staglin Center for Cognitive Neuroscience (Siemens version syngo MR B17) at UCLA. Resting-state functional MR images were collected using a T2*-weighted echo-planar imaging (EPI) sequence with the following parameters: slice thickness = 4 mm, 34 slices, repetition time (TR) = 2 s, echo time (TE) = 30 ms, flip angle = 90°, matrix = 64 × 64, and field of view (FOV) = 192 mm. The resting-state fMRI scan lasted 304 seconds, yielding 152 volumes. Participants were instructed to remain relaxed and keep their eyes open, with no specific stimuli presented and no responses required during the scan. Anatomical MR images were acquired using a T1-weighted high-resolution MPRAGE sequence with the following parameters: slice thickness = 1 mm, 176 slices, TR = 1.9 s, TE = 2.26 ms, matrix = 256 × 256, and FOV = 250 mm. The original UCLA dataset contains 50 participants with schizophrenia and 130 healthy controls. After applying the exclusion criteria, including the removal of participants with overlapping diagnoses and quality control procedures, 44 participants with schizophrenia and 112 healthy controls were included in the final analyses.

The COBRE dataset consists of two groups: individuals diagnosed with schizophrenia (n = 72; mean ± standard deviation: 38.17 ± 14.13) and healthy controls (n = 75; 35.90 ± 11.74), all aged between 18 and 65 years [28]. Exclusion criteria included a history of neurological disorders, intellectual disability, severe head trauma with loss of consciousness exceeding five minutes, and substance use or dependence within the past 12 months. MR images were acquired at the University of New Mexico and the Mind Research Network using a 3T Siemens Trio scanner. Resting-state functional MR images were obtained using a T2*-weighted EPI sequence with the following parameters: slice thickness = 4 mm, 32 slices, TR = 2 s, TE = 29 ms, flip angle = 90°, matrix = 64 × 64, and FOV = 192 mm. The resting-state fMRI scan lasted 300 seconds, yielding 150 volumes. Anatomical MR images were acquired over 6 minutes using a T1-weighted high-resolution MPRAGE sequence with the following parameters: slice thickness = 1 mm, 176 slices, TR = 2.53 s, multiple echo times (5), matrix = 256 × 256, and FOV = 256 mm. Although the COBRE and UCLA datasets were acquired at different sites, they used comparable resting-state fMRI acquisition protocols and were processed using the same standardized preprocessing pipeline, thereby reducing inter-site variability.

A review of the literature indicates that studies employing fMRI and deep learning frameworks to investigate schizophrenia utilize a wide variety of input data representations. Time series derived from functional connectivity analyses, independent component maps, and temporal correlation matrices are among the most commonly used inputs. In contrast, the present study aims to use pre-fMRI images directly as input to deep learning architectures and achieve successful classification without relying on explicitly derived pathological features. Demographic information for participants whose data had completed preprocessing and were used as input to the deep learning models is provided in Table 1.

**Table 1.** **Participants’ demographic information**.

Information	UCLA dataset			COBRE dataset		
	Schizophrenia	Healthy control	*p*-value	Schizophrenia	Healthy control	*p*-value
Participants	44	112	-	34	46	-
Male/Female	33/11	61/51	0.018*	25/9	28/18	0.265
Age range (years), male/female	22–49/26–49	21–49/21–50	-	19–49/20–52	18–62/18–50	-
Mean ± SD	36.41 ± 8.903	31.261 ± 8.736	0.001*	33.345 ± 9.504	35.309 ± 10.986	0.400

**Note 1**: Schizophrenia (total) = 78, Healthy control (total) = 158. SD, standard deviation. For the age range, values are presented as male/female ranges in years. *p*-values for gender were obtained using Pearson’s chi-square test, whereas *p*-values for age were obtained using Welch’s independent-samples *t*-test. * Statistical significance was set at *p* < 0.05. COBRE, Center for Biomedical Research Excellence.

After quality control and preprocessing, a total of 236 participants (78 individuals with schizophrenia and 158 healthy controls) were included in the final analysis. During five-fold cross-validation, stratification was performed according to diagnostic class (schizophrenia/healthy control) to preserve class proportions across folds.

### 2.2 Computer System Specification

The system used in this study consists of an Intel i7-12700 processor with 12 cores and 20 threads operating at a base frequency of 3.6 GHz, 32 GB of 3200 MHz DDR4 RAM, and an NVIDIA RTX 3060 Ti GPU with 8 GB of GDDR6 memory. GPU acceleration was utilized during both the training and inference stages of the deep learning models. For storage, a 512 GB SSD was used for the operating system and active processes, while a 2 TB SATA hard disk was used for data storage. This hardware configuration provided the necessary computational power and data transfer performance, particularly for processing high-dimensional 3D and 4D fMRI data.

### 2.3 Data Preprocessing

The data preprocessing pipeline was performed using the FMRIB Software Library (FSL 6.0.4) (https://fsl.fmrib.ox.ac.uk/fsl/fslwiki). The analyses were performed using Python 3.6.9 (https://www.python.org/) within the Windows Subsystem for Linux (WSL) with Ubuntu 18.04.5 LTS (https://ubuntu.com/wsl) on a Windows 11 operating system. Data stabilization was achieved by discarding the first 7 images from the UCLA and 5 from the COBRE datasets. Preprocessing was then initiated for the remaining 145 images per participant. Preprocessing steps were performed using FSL software to standardize the fMRI data before analysis. First, non-brain tissues were removed using the Brain Extraction Tool (BET) [29]. Subsequently, slice timing correction was applied to align slices acquired at different time points to a reference time point [30]. During the motion correction stage, all volumes were aligned to the middle volume using a 6-degree-of-freedom (3 translations and 3 rotations) linear transformation via the FLIRT algorithm. Participants with a mean head motion exceeding 0.5 mm translation or a maximum head motion exceeding 2 mm translation were excluded [31]. Spatial smoothing was performed using a 5 mm full-width at half maximum (FWHM) Gaussian kernel to enhance the signal-to-noise ratio [32]. For noise removal, independent component analysis-based ICA-AROMA was used to separate motion-related and physiological artifacts from the data [33]. In the temporal filtering stage, a high-pass filter with a cutoff frequency of 0.01 Hz was applied to remove very-low-frequency scanner drift below 0.01 Hz [34]. Finally, functional images were first linearly registered to each subject’s T1-weighted anatomical image using FLIRT [35], and then non-linearly transformed using FNIRT and normalized to the standard MNI152 space, providing a common reference framework for subsequent analyses [36]. Accordingly, following the approaches adopted in the literature [16,37], inter-data variability was minimized to achieve a standardized representation of the data.

### 2.4 Deep Learning-Based Techniques

During this study, a wide range of methods was explored for classifying fMRI data. To improve classification performance for schizophrenia while minimizing overfitting, the final analyses used DarkNet-53, ResNet-50, DarkNet-19, and LSTM-based approaches, and the corresponding methodological details and findings are presented.

DarkNet-53 is a deep neural network architecture developed by Joseph Redmon and serves as the backbone of the YOLOv3 object detection framework [38]. Similar to ResNet, DarkNet-53 incorporates residual connections; however, it is designed to be structurally simpler and more computationally efficient. The architecture consists of 53 convolutional layers and is entirely based on convolutional operations, where down-sampling is primarily achieved through strided convolutions rather than pooling layers. This design provides a balanced trade-off between depth and computational efficiency. While residual blocks facilitate stable gradient flow, the overall structure is simpler than that of ResNet. As a result, DarkNet-53 is well-suited for applications that require both high accuracy and fast processing. It has demonstrated strong performance in image-based classification and detection tasks and has recently been increasingly adopted in medical imaging studies [39,40,41]. The residual connections in DarkNet-53 help mitigate the vanishing gradient problem, enabling effective training of deeper networks. For 3D volumetric data, the DarkNet-53 architecture was adapted by replacing 2D convolutional layers with 3D convolutions, with kernel sizes extended to k × k × k. This modification enabled more effective modelling of spatial context and voxel-wise neighborhood relationships. However, this extension also increased computational cost and GPU memory requirements. Within the scope of this study, DarkNet-53 was applied to 3D ALFF and statistical images, and detailed information on its layers and architecture is presented visually in Fig. 2.

**Fig. 2.**
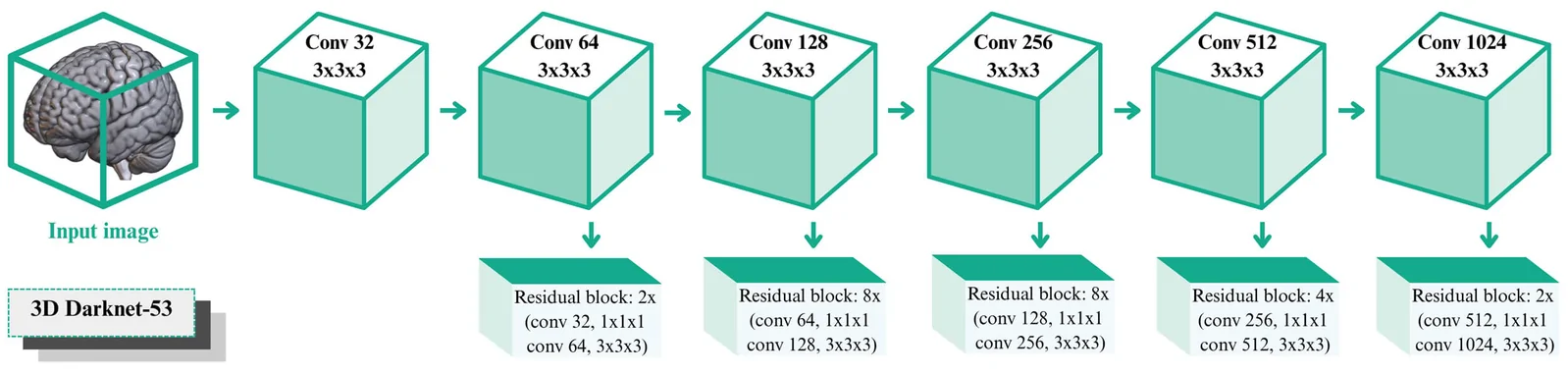
**3D DarkNet-53 architecture**. 3D, three-dimensional.

ResNet-50 is a convolutional neural network model developed by He and colleagues [42], which is widely regarded as a major milestone in the field of deep learning. The primary objective of this architecture is to address performance degradation and vanishing-gradient problems that arise during training very deep networks. The most distinctive feature of ResNet-50 is its use of residual learning. In this approach, instead of directly learning the desired underlying mapping, the network learns the residual between the input and the output. This is achieved through direct connections between layers, known as skip connections. As a result, information and gradient flow can be propagated throughout the network without interruption, enabling the effective training of much deeper architectures. As the name suggests, ResNet-50 consists of 50 layers, and this depth is efficiently managed through specialized bottleneck block structures. The overall architecture includes an initial input layer, followed by a series of residual block groups, and a final fully connected classification layer. Due to its strong performance in transfer learning scenarios, ResNet-50 has been widely adopted in medical imaging applications, particularly for the classification of fMRI and MRI data [18,43]. In this study, the ResNet architecture was extended to 3D by replacing 2D convolutional operations with 3D convolutions and incorporating 3D residual blocks. This approach enables a holistic analysis of brain structures by preserving volumetric information and preventing information loss across slices. However, it also increases training time and may elevate the risk of overfitting. Within the scope of this study, ResNet-50 was applied to 3D ALFF and statistical images, and detailed information regarding its layers and architecture is visually presented in Fig. 3.

**Fig. 3.**
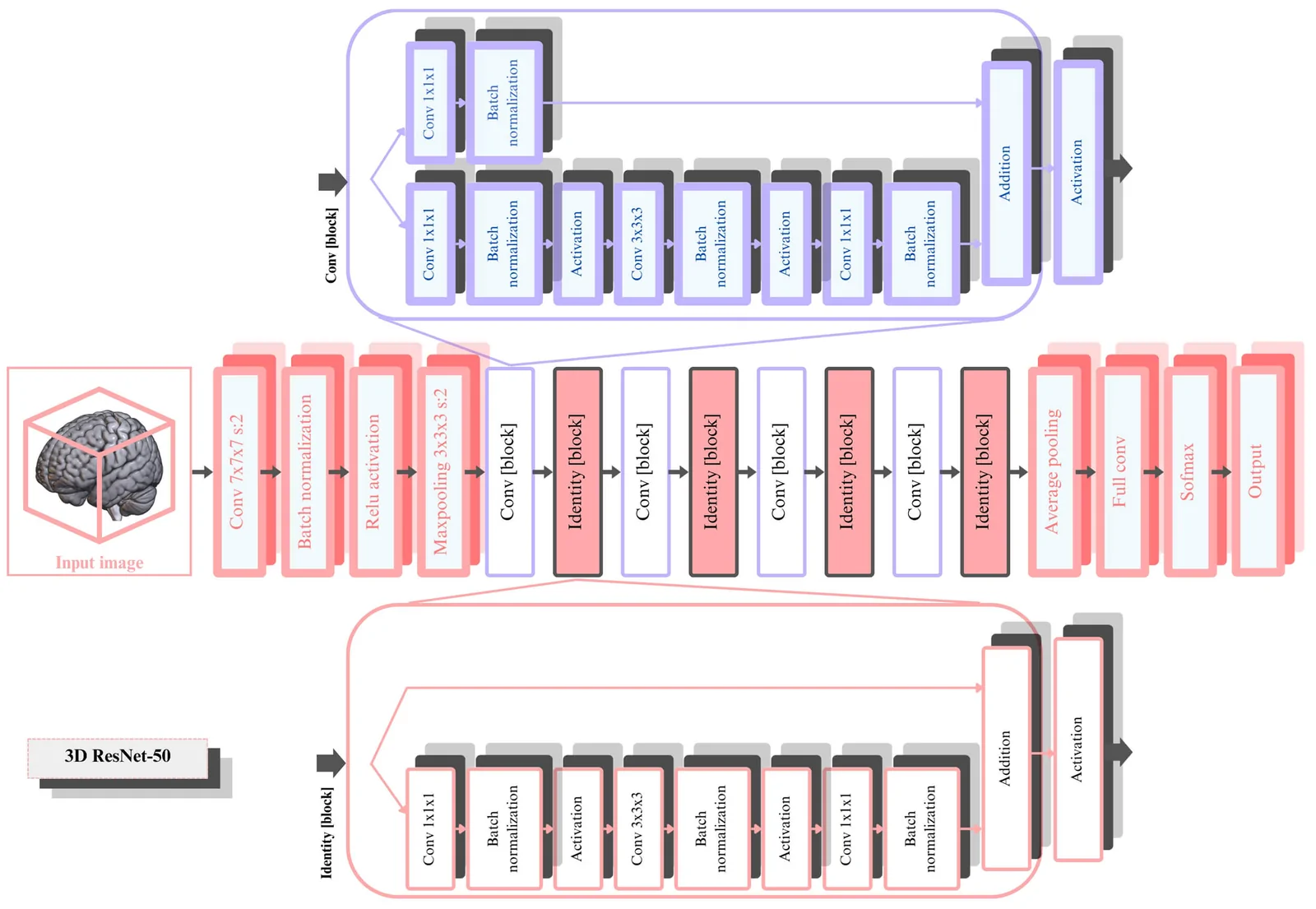
**3D ResNet-50 architecture**.

In conclusion, while ResNet-50 excels at learning deeper, more expressive feature representations, DarkNet-53 offers a faster, more computationally efficient alternative. The combined or comparative use of these two architectures can provide significant advantages in both accuracy and efficiency, particularly for the analysing high-dimensional, complex fMRI data. LSTM is a type of RNN developed to learn long-term dependencies in time series data [44]. Since fMRI data inherently contain both spatial and temporal information (4D: x, y, z, t), LSTM models are particularly well-suited to this data structure. Within the 4D spatiotemporal framework, DarkNet-53 was initially considered for spatial feature extraction. However, it was observed that this architecture could not be efficiently executed on the available system due to its high computational cost and memory requirements. In particular, processing 3D volumes at each time step within a 4D data structure significantly increased GPU memory usage and caused computational strain during training. Therefore, to achieve a more stable and sustainable training process, a lighter and more computationally efficient architecture, DarkNet-19, was selected [45]. The reduced depth and fewer parameters in DarkNet-19 enabled the 3D + LSTM hybrid framework to operate within system resource constraints. This modification allowed uninterrupted model training and the successful completion of experiments focused on learning temporal dependencies. While this approach enables the integration of both spatial and temporal information, it still requires substantial computational resources.

### 2.5 Experimental Design

In this study, a comprehensive, systematic experimental design was conducted to discriminate between individuals with schizophrenia and healthy controls using different data representations (3D and 4D) and deep learning architectures. The primary aim of the study is to examine, within a holistic framework, not only the impact of model architecture but also the effect of data representation on classification performance. Data representation is one of the most critical components directly influencing the success of deep learning models, and minimizing information loss is particularly important in high-dimensional neuroimaging data. Accordingly, the effects of increasing data dimensionality, preserving spatial and temporal information, and applying different feature extraction strategies on model performance were analysed in detail. In addition, the practical computational feasibility of each experimental setup was considered alongside classification performance. To improve clarity and facilitate understanding of the experimental design, the fMRI image representations used as inputs to the subject-level deep learning models under the different methodological frameworks are illustrated in Fig. 4.

**Fig. 4.**
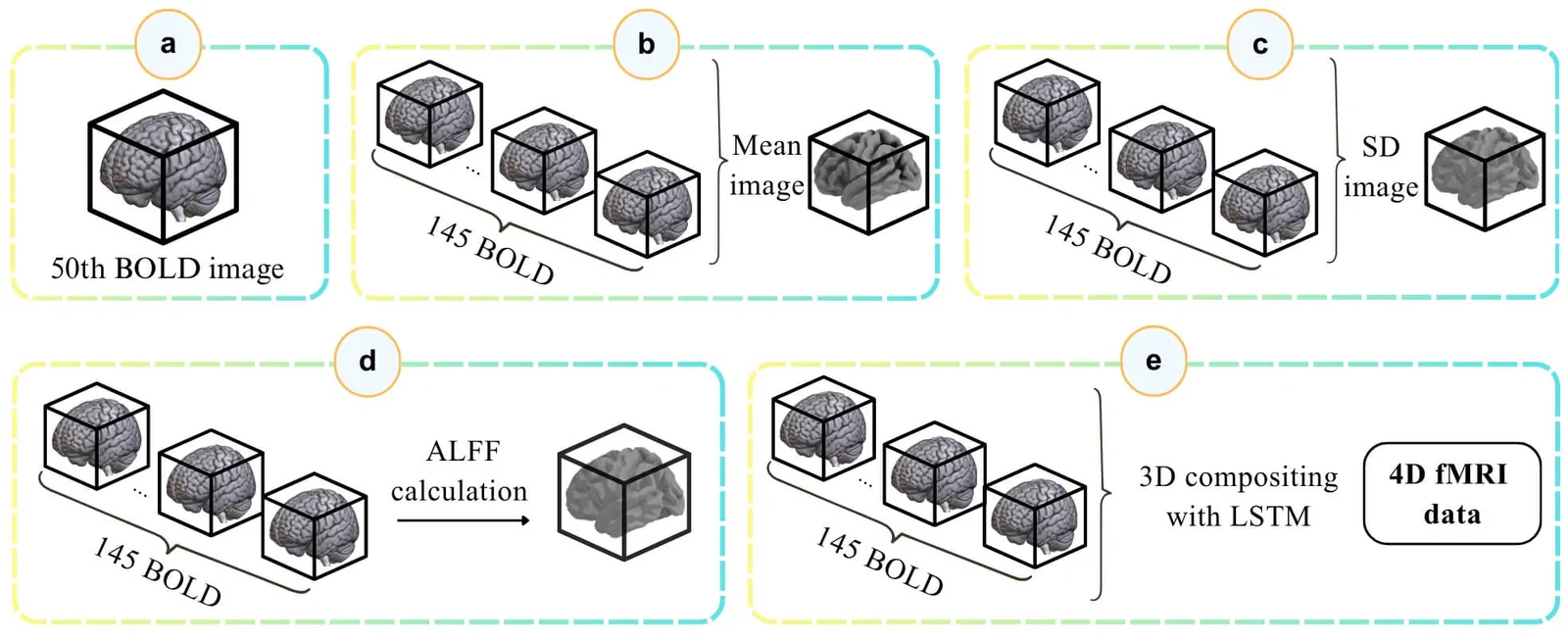
**Image representations generated from resting-state fMRI data and used as inputs to the deep learning models**. (a) 50th BOLD volume, used as the baseline static representation. (b) Mean BOLD image. (c) Standard deviation (SD) BOLD image. (d) Amplitude of Low-Frequency Fluctuation (ALFF) image. (e) Four-dimensional (4D) resting-state fMRI image preserving both spatial and temporal information. Abbreviations: fMRI, functional magnetic resonance imaging; BOLD, blood oxygen level-dependent; LSTM, Long Short-Term Memory.

#### 2.5.1 3D Volumetric Approach

In the first approach (Fig. 4a), fMRI data corresponding to the 50th BOLD volume were directly fed into the model as three-dimensional volumetric data, without any slicing. In this way, the brain’s spatial structure and interregional relationships were preserved, aiming to obtain a more holistic representation. Using 3D convolutional layers, the model was enabled to learn not only two-dimensional local features but also volumetric contextual information. The initial volumes of the fMRI acquisition were discarded to minimize the potential effects of transient signal instability during the early phase of data acquisition [46]. In contrast, later volumes may be more susceptible to cumulative motion and physiological fluctuations. Consequently, the 50th volume corresponds to the middle portion of the acquisition, where signal stabilization is expected after the initial transient period while still preserving representative spontaneous BOLD activity. The 50th BOLD volume was therefore included as a baseline representation to facilitate a direct comparison with the derived image representations evaluated in this study. Nevertheless, this selection is empirical, and future studies may investigate multiple representative volumes or ensemble strategies.

In the second approach (Fig. 4b,c), mean and standard deviation maps were generated to represent temporal information in a more compact and informative manner. In this framework, mean 3D maps reflect the overall level of brain activity, whereas standard deviation maps represent temporal variability. These two representations were separately input into the models to evaluate which statistical feature provides greater discriminative power. This approach offers a significant advantage in transforming high-dimensional time series data into a more manageable form. Across these representations, the contributions of both static and dynamic features to classification performance were compared. The main advantage of the 3D approach lies in its ability to preserve the spatial integrity of brain activity, enabling the learning of more meaningful features. However, its drawbacks, such as increased computational cost and model complexity, were also taken into consideration.

In the third approach (Fig. 4d), 3D data representations were constructed using ALFF maps derived from multidimensional fMRI data. ALFF measures the amplitude of low-frequency BOLD signal fluctuations and serves as an important biomarker reflecting spontaneous brain activity [47]. ALFF values were then computed voxel-wise using a custom Python implementation based on the original ALFF methodology. The preprocessed BOLD time series (145 volumes) was transformed into the frequency domain using the Fast Fourier Transform (FFT), and the amplitude spectrum (i.e., the square root of the power spectrum) was averaged within the low-frequency range of 0.01–0.08 Hz to generate the ALFF map [47]. Finally, each ALFF map was normalized by its global mean ALFF value (mALFF) before being used as input to the deep learning models. The ALFF computation was performed using the complete resting-state BOLD time series (145 volumes per participant), yielding a single 3D ALFF map that summarizes spontaneous low-frequency brain activity. The resulting ALFF maps were directly used as inputs to the deep learning models, and their contribution to classification performance was systematically analysed.

#### 2.5.2 4D Approach (3D Spatial + 1D Temporal)

Due to the nature of the fMRI data used in this study, which inherently contains both spatial and temporal information, a four-dimensional data representation was also evaluated. In this approach (Fig. 4e), the aim is to incorporate not only spatial features but also temporal variations into the model. Accordingly, a hybrid model architecture was constructed. Spatial features were extracted from each time point’s 3D volume using DarkNet-19. This model was preferred over DarkNet-53 due to its lower computational cost. Temporal dependencies among these extracted features were then learned using LSTM. In the proposed architecture, each time-specific 3D volume was first processed through 3D CNN layers, and the resulting feature vectors were subsequently fed sequentially into the LSTM network. This structure enables the learning of temporal evolution patterns and their contribution to classification performance. This approach is consistent with widely used CNN + RNN/LSTM hybrid models reported in the literature [11,14] and is considered a powerful method for analysing time-series data. However, factors such as increased model complexity, longer training time, and the risk of overfitting were also considered during the experimental evaluations.

### 2.6 Model Training and Validation

To ensure a reliable and generalizable evaluation of model performance across all experiments while preventing data leakage and providing an unbiased assessment on unseen data, a five-fold cross-validation strategy was employed. No oversampling, undersampling, or class-weighting strategy was applied during training. To preserve class balance, a stratified five-fold cross-validation based on diagnostic labels was adopted. No stratification according to acquisition site was applied. In this approach, the dataset was divided into five equal subsets, with each iteration using four subsets for training and one for testing. For each fold, the model was retrained independently, and the resulting accuracy values were averaged to obtain the overall performance. This strategy was adopted to assess the model’s consistency across different data splits, reduce the risk of overfitting, and obtain more robust performance metrics. In addition, careful attention was paid to data partitioning throughout the experimental process to preserve sample independence. This ensured that the obtained results could be more effectively generalized to real-world applications. All methodological approaches and the overall experimental pipeline developed in this study are illustrated in Fig. 5 to provide a comprehensive overview of the process. To evaluate whether the differences in classification performance among the 3D CNN models and data representations were statistically significant, pairwise statistical comparisons were conducted using the fold-wise cross-validation results. As the 4D spatiotemporal model differed fundamentally in both data representation and network architecture, it was not included in these comparisons. Depending on the normality of the paired differences, either paired *t*-tests or Wilcoxon signed-rank tests were applied. Effect sizes and 95% confidence intervals (where applicable) were also calculated to quantify the magnitude of the observed differences. The detailed statistical results are presented in **Supplementary Table 1**.

**Fig. 5.**
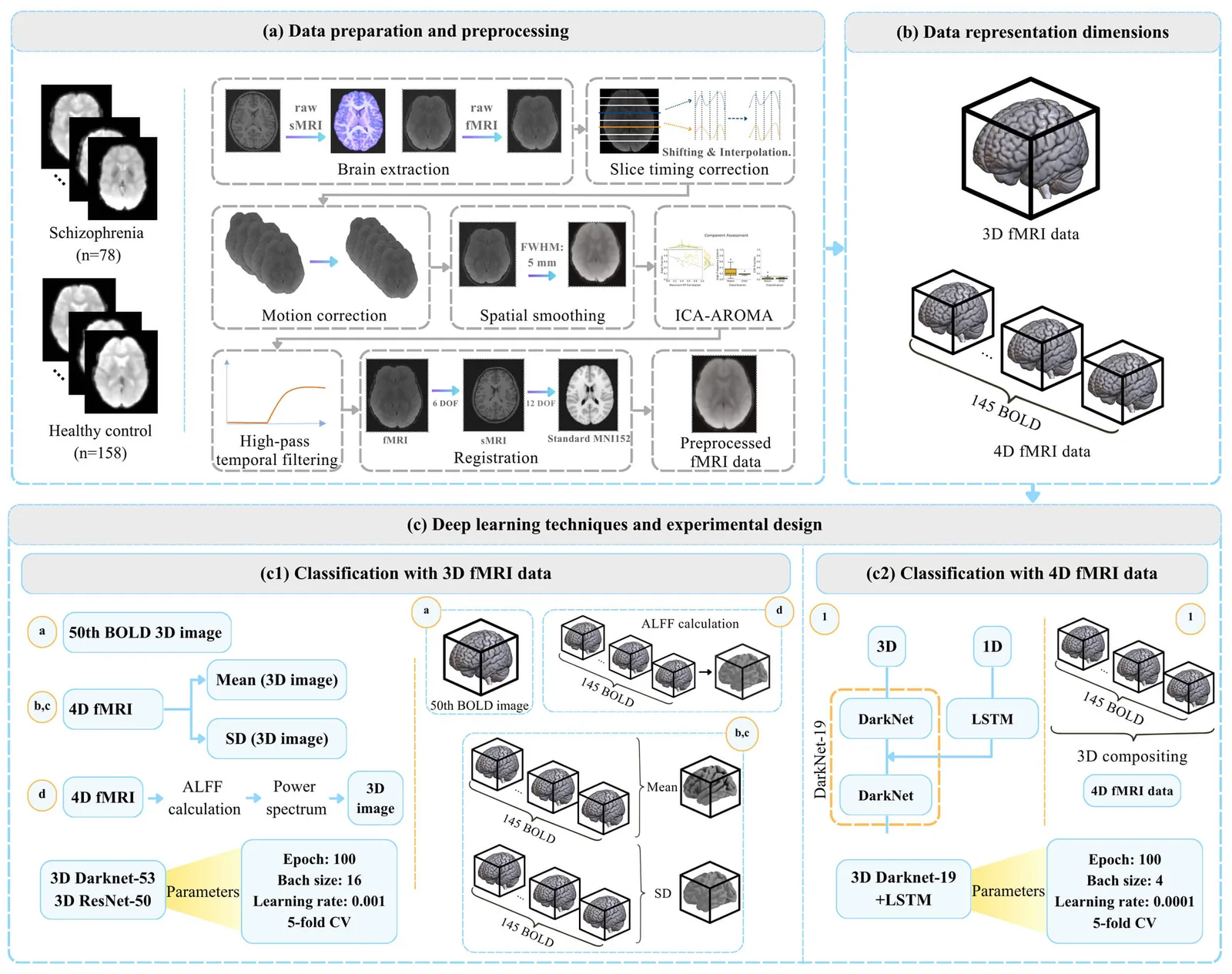
**Overview of the proposed experimental workflow for deep learning-based schizophrenia classification using resting-state fMRI data**. (a) Preprocessing and preparation of the resting-state fMRI data. (b) Dimensions of the evaluated fMRI data representations, including 3D and 4D representations. (c1) Classification pipeline based on the 3D image representations using the evaluated CNN architectures. (c2) Classification pipeline based on the 4D spatiotemporal representation using the CNN–LSTM architecture. Abbreviations: fMRI, functional magnetic resonance imaging; BOLD, blood oxygen level-dependent; SD, standard deviation; ALFF, amplitude of low-frequency fluctuation; LSTM, long short-term memory.

The hyperparameter configurations evaluated during model development are summarized in Table 2. During hyperparameter tuning, batch sizes of 16 and 32 were evaluated for the 3D models, whereas 2, 4, and 8 were explored for the 4D model due to its higher computational requirements. In addition, the 4D model was trained for 100 to 150, 250, and 500 epochs during optimization. The final hyperparameter values adopted for each model are presented in Table 2. Different hyperparameter settings were used for the 3D and 4D models due to their substantially different computational requirements. The 4D spatiotemporal model processes an additional temporal dimension, resulting in considerably higher GPU memory consumption and computational complexity than the 3D models. Therefore, different hyperparameter settings were employed for the 4D model due to its substantially higher computational requirements, whereas the same optimization strategy was maintained across all 3D models to ensure a fair comparison.

**Table 2.** **Hyperparameter settings used for model training**.

Hyperparameter	3D (50th/Mean/SD Image Models)	3D (ALFF Model)	4D (DarkNet-3D + LSTM)
Batch size	16	16	4
Number of epochs	100	100	100
Learning rate	0.001	0.001	0.0001
Loss function	Cross-Entropy Loss	Cross-Entropy Loss	Cross-Entropy Loss
Optimizer	Adam	Adam	Adam

**Note 2**: SD, Standard deviation; ALFF, Amplitude of Low-Frequency Fluctuations; LSTM, Long Short-Term Memory.

## 3. Results

In this study, the classification of schizophrenia and healthy control groups was evaluated comprehensively and comparatively using different data representations (3D and 4D) and deep learning architectures. Across all experimental procedures, a five-fold cross-validation strategy was used to enhance model generalizability and minimize overfitting. In addition to fold-wise results, average performance metrics were also computed for each model. The findings demonstrate that both data representation and model architecture play a decisive role in classification performance. The demographic characteristics of the participants are presented in Table 1. Statistical analyses showed significant differences in both age (*p* = 0.001) and gender distribution (*p* = 0.018) between schizophrenia and healthy controls in the UCLA dataset. In contrast, neither age (*p* = 0.400) nor gender distribution (*p* = 0.265) differed significantly between groups in the COBRE dataset.

Overall, the results indicate that the highest classification performance was achieved with ALFF-based 3D data representations. In this context, ResNet-50 achieved an average accuracy of 80.10%, an AUC of 75.95%, and an F1-score of 86.06%. For the same data representation, DarkNet-53 demonstrated a comparable performance with 79.22% accuracy, 76.92% AUC, and 85.71% F1-score. These results suggest that low-frequency BOLD signal components contain discriminative information relevant to schizophrenia and provide a strong representation for deep learning models.

### 3.1 3D Classification Results

In classifications performed on the raw 3D BOLD volume (50th BOLD image), the average accuracies obtained with DarkNet-53 and ResNet-50 were 73.76% and 77.11%, respectively. The corresponding average AUC values were 63.65% and 73.22%, while the average F1-score values were 82.72% and 84.59%. The higher performance of the ResNet-50 model across both AUC and accuracy indicates that deeper residual architectures can learn more discriminative features.

When 3D representations based on the mean BOLD signal were examined, the DarkNet-53 model achieved 72.45% accuracy, 63.99% AUC, and 81.89% F1-score, whereas the ResNet-50 model reached 75.84% accuracy, 73.09% AUC, and 82.60% F1-score. These results suggest that statistical summary representations provide some discriminative information, though they are less informative than ALFF-based representations. In the standard deviation-based 3D analyses, noteworthy results emerged. The DarkNet-53 model achieved 79.22% accuracy, 76.49% AUC, and 86.18% F1-score, while the ResNet-50 model yielded 73.74% accuracy, 59.83% AUC, and 83.21% F1-score. In particular, DarkNet-53’s superior performance on this data representation suggests that certain architectures may better capture temporal signal variability.

In analyses using ALFF-based 3D data representations, generally superior results were obtained across the experiments. The ResNet-50 model achieved the highest performance with an average accuracy of 80.10%, an AUC of 75.95%, and an F1-score of 86.06%, while the DarkNet-53 model achieved 79.22% accuracy, 76.92% AUC, and 85.71% F1-score. The high F1-score values obtained by both models indicate that classification was performed in a balanced manner. Overall, the 3D analyses indicate that the ResNet-50 architecture generally achieves higher accuracy and AUC across most data representations, whereas DarkNet-53 can produce competitive and even superior results, particularly for standard deviation-based representations. These findings highlight that the interaction between data representation and model architecture is critical to classification performance. Detailed results for single-volume, ALFF-based, standard deviation-based, and mean-based analyses are presented in Table 3.

**Table 3.** **Classification results obtained from 3D and 4D fMRI images used as input to deep learning models**.

Models	Input data	Data size	Mean AUC (%)	Best AUC (%)	Mean accuracy (%)	Best accuracy (%)	Mean F1 (%)	Best F1 (%)
DarkNet-53	50th bold image (Fig. 4 (a))	3D	63.65	69.58	73.76	78.72	82.72	86.11
ResNet-50			73.22	79.69	77.11	80.85	84.59	86.96
DarkNet-53	Mean image (Fig. 4 (b))	3D	63.99	76.76	72.45	76.60	81.89	84.93
ResNet-50			73.09	79.48	75.84	80.85	82.60	87.32
DarkNet-53	SD image (Fig. 4 (c))	3D	76.49	90.32	79.22	85.11	86.18	89.23
ResNet-50			59.83	69.17	73.74	78.72	83.21	86.11
DarkNet-53	ALFF Image (Fig. 4 (d))	3D	76.92	85.83	79.22	83.33	85.71	88.89
ResNet-50			75.95	86.88	80.10	87.23	86.06	90.32
DarkNet-19 +LSTM	3D spatial + 1D temporal (Fig. 4 (e))	4D	73.56	81.25	76.68	80.85	83.96	86.96

**Note 3**: AUC, Area Under the Curve; ALFF, Amplitude of Low-Frequency Fluctuation; SD, Standard deviation; LSTM, Long Short-Term Memory.

Pairwise statistical comparisons demonstrated that, among the 3D CNN models, the ALFF representation significantly outperformed the mean image representation for DarkNet-53 in terms of both accuracy (*p* = 0.001) and F1-score (*p* = 0.035). For ResNet-50, ALFF achieved significantly higher accuracy than the standard deviation representation (*p* = 0.040). In contrast, no significant differences were observed between DarkNet-53 and ResNet-50 when the same image representation (including ALFF) was used (all *p* > 0.05), suggesting that image representation had a greater influence on classification performance than the CNN architecture. Detailed statistical comparisons are provided in **Supplementary Table 1**.

### 3.2 4D Classification Results

In analyses that used a four-dimensional data structure incorporating the temporal dimension, a hybrid approach integrating a 3D DarkNet-19 architecture with LSTM layers was employed to model spatial and temporal information jointly. This modelling strategy aimed to capture the inherent temporal dependencies present in fMRI data. The obtained results indicate that the 4D hybrid model achieved an average accuracy of 76.68%, an average AUC of 73.56%, and an average F1-score of 83.96%. These findings suggest that the model provides a generally balanced and consistent classification performance. However, it is noteworthy that, contrary to expectations, the 4D models did not yield a substantial performance improvement compared to the 3D approaches. Although the 4D framework achieved its highest single-fold accuracy (80.85%) and AUC (81.25%) within the five-fold cross-validation, its average cross-validation performance was lower, indicating that these high values were not consistently maintained across the folds. This finding suggests that while spatiotemporal modelling has considerable potential, its performance may be more sensitive to model capacity and to limited sample sizes. The main reasons for this observation may include increased model complexity, longer training time, difficulties with parameter optimization, limited dataset size, and insufficient ability to capture temporal patterns. In addition, LSTM layers typically require longer and richer time series to operate effectively, and the available data structure may not fully support this requirement. The fold-based results nevertheless indicate that the model exhibits relatively consistent learning behavior. However, when evaluated in terms of overall performance, the gain achieved does not appear to justify the increased computational cost. The results obtained using the 4D data representation are presented in detail in Table 3.

## 4. Discussion

The findings of this study clearly demonstrate that, in schizophrenia classification using resting-state fMRI data, both the choice of data representation and the selected deep learning architecture play a decisive, interacting role in performance. This indicates that focusing solely on algorithmic improvements is insufficient and that the data structure itself is a critical factor. The results further suggest that not only model selection, but also the biological relevance and representational form of the input data are equally important as the architecture itself. Therefore, strategies to improve model performance should be considered alongside data representation. When evaluated alongside the literature, it is observed that data representations reflecting functional brain activity, either directly or indirectly, tend to provide higher discriminative power, and the findings of this study are largely consistent with this trend.

In our study, the highest classification performance was achieved using ALFF-based 3D data representations and the ResNet-50 architecture (80.10%), suggesting that low-frequency BOLD signal fluctuations contain diagnostically relevant information for schizophrenia classification. Since ALFF directly reflects the amplitude of spontaneous neural activity, it has a high capacity to capture disease-related neurophysiological alterations, making it a biologically meaningful representation for deep learning-based classification. This observation is consistent with studies reported in Table 4 (Ref. [10,11,12,13,14,15,16,17,18,19,37,43,48,49]), which highlight voxel-based activity maps as powerful biomarkers. In particular, the work by Ghanbari et al. [14], which achieved high accuracy by jointly using multiple activity measures, demonstrates the strong discriminative capability of activity-based representations and further suggests that combining complementary measures may enhance classification performance. Similarly, another study [50] reported approximately 79% classification accuracy using resting-state functional connectivity analyses, further supporting the clinical relevance of low-frequency BOLD signal fluctuations. The consistency between our findings and previous studies strengthens the validity of the proposed ALFF-based representation. The superior performance of ALFF observed in this study may be explained by its ability to quantify spontaneous low-frequency (0.01–0.08 Hz) BOLD oscillations, which are widely regarded as markers of intrinsic neuronal activity during the resting state [51]. Previous neuroimaging studies have consistently demonstrated that schizophrenia is associated with abnormal low-frequency fluctuations across several large-scale brain networks, including the default mode, salience, frontoparietal, auditory, and thalamocortical networks [52,53]. These alterations are thought to reflect disrupted neural synchronization, impaired excitatory-inhibitory balance, and abnormal functional integration, all of which are core neurobiological characteristics of schizophrenia [54,55]. Because ALFF directly measures the amplitude of these spontaneous oscillations, it is likely to preserve diagnostically relevant physiological information more effectively than statistical representations such as mean, standard deviation, or a single BOLD snapshot. Consequently, ALFF-based representations may provide richer disease-related information for convolutional neural networks, thereby contributing to the superior classification performance observed in this study.

**Table 4.** **Summary of functional MRI-based schizophrenia classification studies**.

References (Authors, year)	Dataset	Data group size (SCZ/HC)	Image size	Age scale	Features of input data	Classification techniques	Findings
Task-based fMRI studies							
Oh et al., (2019) [10]	---	103/41	3D activation maps	18–59	-3D activation map	3D CAE-CNN	84.43% accuracy 88.42% sensitivity 80.06% specificity
Dakka et al., 2017 [11]	FBIRN	46/49	3D volumetric fMRI	---	-Preprocessed 4D fMRI image	LSTM R-CNN	SVM: 57.89% accuracy R-CNN and LSTM: 65% accuracy
Resting-state fMRI studies							
Raeisi et al., 2025 [18]	UCLA Openneuro	83/75	2D fMRI slice	20–45	-Normalization -Noise reduction -Contrast enhancement	VGG16, ResNet-50, Xception, SVM, RF, GB	CNN-based approach 98.67% accuracy ResNet-50: 98.71% accuracy SVM: 97.01%, RF: 98.44%, GB: 98.84% accuracy
Goodarzi et al., 2025 [19]	Publicly available dataset	25/12	Slice-wise 2D image	20–44	-Normalization -Noise reduction -Contrast enhancement	CNN CSVM CRF CGB	CNN: 99.99% accuracy CSVM: 99.80% accuracy CRF: 99.82% accuracy CGB: 99.84% accuracy
Wang et al., 2024 [12]	Xinxiang Medical College, COBRE	88/81	2D correlation matrix	18–55	-116 regions in the AAL atlas -Pearson correlation -Spearman correlation -FC matrix	MSCN	Special dataset: 91.71% accuracy, 94.99% specificity, 88.69% sensitivity, 91.82% F1 score, 91.84% AUC COBRE dataset: 79.62% accuracy, 78.52% sensitivity, 79.71% specificity, 79.61% AUC
Ghanbari et al., 2022 [14]	COBRE	72/74	3D ALFF, fALFF, ReHo, VMHC maps	24–52	-ReHo analysis -ALFF analysis -fALFF analysis -VMHC feature maps	3D CNN -LSTM	3D CNN: 87.63% accuracy 3D CNN-LSTM: 88.15% accuracy Combined features: 3D CNN: 90.91% accuracy 3D CNN-LSTM: 92.32% accuracy
Yan et al., 2022 [13]	multi-site B-SNIP	176/229	Time series	24–50	-Time series of five independent components	SVM RF AdaBoost MsRNN	MsRNN: SZ & HC: 78.5% accuracy
Lei et al., 2022 [15]	6 different datasets	505/907	FC matrix	15–53	-90 anatomical regions according to the AAL atlas -FC matrix -Correlation coefficient, z-score with Fisher r-to-z	GCN	10-fold CV: GCN: 85.8% accuracy, 92.6% AUC; SVM: 80.9% accuracy, 89.7% AUC LOSO: GCN: 79.1% accuracy, 79.0% AUC; SVM: 73.4% accuracy, 75.3% AUC 160 brain regions: GCN: 83.7% accuracy, SVM: 75.3% accuracy
Zheng et al., 2021 [43]	COBRE	98/102	3D fMRI data	18–65	-Preprocessed data -Time series inference -Correlation on ROI regions	AlexNet VGG16 ResNet-50	87.85% accuracy, 87.11% sensitivity, AlexNet: 78.36% accuracy, VGG16: 85.27% accuracy ResNet-50: 83.09% accuracy
Hashimoto et al., 2021 [49]	COBRE	69/72	3D fMRI volume	23–52	-MRI volumes obtained from each time point	Supervised deep learning	Proposed features: 61.1% accuracy, Classification + identification features: 77.8% accuracy, FC matrix: 72.2% accuracy
Zhao et al., 2021 [16]	7 different centers	558/542	Time series (1D), FC (2D)	18–45	-FC matrix of independent components	GAN	10-fold CV: DNN: 80.3% accuracy, GAN: 82.1% accuracy LOOCV: DNN: 77.0% accuracy, GAN: 80.7% accuracy
Lei et al., 2020 [37]	5 different centers	295/452	4D, 2D FC matrix	23–53	-Preprocessed whole-brain fMRI images -Connectome matrix -Graph-based analytical metrics	Deep learning LR SVM	FC features: LR: 80.97%, SVM: 81.74%, DL: 81.03% accuracy Graph-based metrics: LR: 68.25%, SVM: 72.00%, DL: 68.61% accuracy
Qureshi et al., 2019 [48]	COBRE	72/72	3D ICA maps	23–50	-Fifteen independent components	3D CNN	98.09% accuracy, 99.82% AUC, 98.62% specificity, 97.49% sensitivity
Yan et al., 2019 [17]	7 different centers	558/542	ROI, ICA-based time series (1D)	18–45	-Time series of fifty independent components	CRNN	Multi-site pooling: 83.2% accuracy Leave-one-site: 80.2% accuracy
Proposed study	COBRE, UCLA	78/158	3D and 4D fMRI image	18–62	-Pre-fMRI data -ALFF-based 3D volumes -Statistical summary representations (mean, SD)	DarkNet-53 ResNet-50 DarkNet-19 LSTM	50th bold: ResNet-50: 77.11%, DarkNet-53: 73.76% ALFF: ResNet-50: 80.10%, DarkNet-53: 79.22% Mean bold: ResNet-50: 75.84%, DarkNet-53: 72.45% SD bold: ResNet-50: 73.74%, DarkNet-53: 79.22% 4D fMRI: DarkNet-19 + LSTM: 76.68% *All results given represent accuracy metrics.

**Note 4**: SCZ, Schizophrenia; HC, Healthy control; MRI, Magnetic Resonance Imaging; sMRI, Structural Magnetic Resonance Imaging; fMRI, Functional Magnetic Resonance Imaging; ALFF, Amplitude of Low-Frequency Fluctuations; fALFF, Fractional Amplitude of Low-Frequency Fluctuations; ReHo, Regional Homogeneity; VMHC, Voxel-Mirrored Homotopic Connectivity; ROI, Region of Interest; ICA, Independent Component Analysis; sFNC, Static Functional Network Connectivity; FC, Functional Connectivity; CNN, Convolutional Neural Network; 3D CNN, Three-Dimensional Convolutional Neural Network; LSTM, Long Short-Term Memory; RNN, Recurrent Neural Network; CRNN, Convolutional Recurrent Neural Network; SAE, Stacked Autoencoder; CAE, Convolutional Autoencoder; GCN, Graph Convolutional Network; GAN, Generative Adversarial Network; DNN, Deep Neural Network; MSCN, Multiple Sparsely Connected Network; MultiViT, Multimodal Vision Transformer; Grad-CAM, Gradient-weighted Class Activation Mapping; SVM, Support Vector Machine; k-NN, k-Nearest Neighbors; RF, Random Forest; GB, Gradient Boosting; LR, Logistic Regression; AUC, Area Under the Curve; DWT, Discrete Wavelet Transform; AAL, Automated Anatomical Labeling; COBRE, Center for Biomedical Research Excellence; FBIRN, Function Biomedical Informatics Research Network; B-SNIP, Bipolar-Schizophrenia Network on Intermediate Phenotypes; SD, Standard Deviation.

Although several studies in Table 4 report high accuracy values using 3D CNN-based approaches [10,14,43,48], they often rely on either different dataset sizes or pre-selected meaningful feature maps. Therefore, direct comparisons should be interpreted cautiously, considering methodological differences. On the other hand, slice-wise 2D approaches using transfer learning have reported accuracies reaching up to 98% [18,19]. However, such high accuracy values are frequently discussed in the literature as potentially subject to methodological limitations, including data leakage, slice dependency, and violations of sample independence. In this regard, it should be emphasized that high accuracy does not necessarily imply high generalizability.

In 4D approaches that incorporate the temporal dimension (3D + LSTM), the lack of the expected performance improvement is a notable finding. This result clearly demonstrates that the relationship between model complexity and performance is not linear. Although fMRI data exhibit temporal dependencies, effectively capturing them requires larger datasets and more sophisticated optimization strategies. Otherwise, models may struggle to learn meaningful temporal patterns from the available data. The literature in Table 4 also reports that LSTM or RNN-based approaches provide only limited improvements over conventional methods [11,13]. Similarly, in our study, the performance of the 4D model remained around 76%, indicating that while it sometimes outperforms certain 3D configurations, increased model complexity does not always translate into improved performance. Additionally, high-dimensional data structures increase the risk of overfitting and make training more challenging, which further explains these results. This clearly shows that increasing data dimensionality alone is not a sufficient strategy. Furthermore, in 4D fMRI analysis, temporal information was incorporated through an LSTM network, consistent with established methodologies reported in the literature. Importantly, this observation should not be interpreted as evidence that spatiotemporal modelling is inherently inferior. Because computational limitations required replacing DarkNet-53 with the lighter DarkNet-19 backbone, differences in representational capacity may also have contributed to the lower average performance. Moreover, the relatively small performance gap between the 3D and 4D models suggests that the additional temporal modelling provided only limited benefits under the present experimental conditions. The increased variability observed in the 4D analysis may be attributed to the complexity of modelling temporal dynamics, as well as to the greater sensitivity of sequence-based learning to temporal and inter-subject variations in resting-state fMRI data. Given the substantially higher computational cost of processing 4D data, the proposed 3D ALFF-based framework offers a more practical, computationally efficient alternative while maintaining competitive classification performance.

The observation that standard deviation-based statistical maps outperform mean-based maps suggests that temporal variability may be an important discriminative feature in schizophrenia. This finding is one of the most novel contributions of the study. It is also consistent with previous literature reporting the importance of dynamic connectivity changes in functional brain networks. Studies [12,56] have shown that functional connectivity patterns and network dynamics significantly contribute to classification performance. Therefore, the results emphasize the importance of considering not only static but also dynamic brain characteristics. Standard deviation maps may preserve moment-to-moment variability of spontaneous BOLD activity, reflecting unstable neural dynamics and altered brain state transitions that have previously been associated with schizophrenia through dynamic functional connectivity and neural metastability studies [57,58,59]. The relatively low AUC despite acceptable classification accuracy observed for the SD-based ResNet model illustrates that these metrics quantify different aspects of model performance. Accuracy reflects predictions at a fixed threshold, whereas AUC evaluates ranking ability across all thresholds, which can lead to such discrepancies, particularly in modest-sized datasets.

The additional statistical comparisons further supported the importance of image representation in CNN-based schizophrenia classification. Among the 3D models, the ALFF representation demonstrated significantly higher classification performance than the mean image representation for DarkNet-53 and higher accuracy than the standard deviation representation for ResNet-50. In contrast, no significant performance differences were observed between DarkNet-53 and ResNet-50 when the same image representation was used, except for the mean image representation. These findings suggest that biologically meaningful image representations may have a greater influence on classification performance than the choice of CNN architecture within conventional 3D models.

One of the key contributions of this study is the direct use of pre-fMRI images as input to deep learning models, rather than the commonly used functional connectivity matrices, ICA components, or ROI-based time series. This approach simplifies the processing pipeline by providing an end-to-end image-based learning framework for both minimally processed (50th bold, Mean and SD image) and biologically derived (ALFF) image representations. While most studies in the literature rely on derived features obtained after connectivity analysis [15,16], this study aims to perform classification using representations closer to raw imaging data. This approach eliminates additional computational steps required for connectivity analysis and offers a more practical framework. However, this simplicity may also limit the explicit modelling of complex functional relationships. Moreover, the relatively lower accuracy compared to some connectivity-based studies suggests that explicitly modelled functional relationships can improve classification performance.

From a model architecture perspective, ResNet-50 demonstrated higher and more stable performance than DarkNet-53. This may be related to the use of residual connections, which facilitate the learning of deep feature representations by helping to mitigate the vanishing gradient problem [60]. This finding is consistent with studies reporting strong performance of ResNet-based models in the literature [18,43]. On the other hand, DarkNet-53 achieved competitive results at a lower computational cost, making it a suitable alternative for resource-constrained applications. This highlights that computational efficiency should also be considered alongside performance when selecting models.

From a clinical perspective, the present findings suggest that biologically meaningful image representations, particularly ALFF maps, may improve the performance of deep learning models for schizophrenia classification while maintaining a relatively simple analysis pipeline [14,61,62]. Rather than serving as standalone diagnostic systems, such approaches may ultimately contribute to computer-aided decision-support tools that complement clinical assessment by providing objective neuroimaging-based information [62,63,64]. Nevertheless, the current classification performance, although encouraging, remains insufficient for routine clinical application and should be interpreted within the context of a proof-of-concept study. Furthermore, despite combining two publicly available multi-site datasets, the proposed framework was not validated using an independent external cohort, which may limit its generalizability across different imaging centers and patient populations. The interpretation of the present findings should also consider that potentially important clinical variables, such as medication status and illness duration, were not incorporated into the analysis. Although the COBRE and UCLA datasets provide well-characterized diagnostic information, these clinical variables were not consistently available for all participants and therefore could not be evaluated. As both medication exposure and disease chronicity may influence resting-state brain activity, future studies using larger, prospectively collected multi-center cohorts with comprehensive clinical characterization are needed to validate the proposed framework and further investigate its potential clinical utility [7].

Overall, this study demonstrates, in agreement with the literature, that data representation may play a more critical role than model architecture in schizophrenia classification. This conclusion provides an important direction for future research. In particular, the use of biologically meaningful features, such as ALFF, improves performance, whereas simply increasing data dimensionality does not necessarily yield the expected gains. These results suggest that future studies may benefit from hybrid approaches combining both activity- and connectivity-based features, as well as from larger, more balanced datasets. Furthermore, improving model interpretability and identifying discriminative brain regions would provide valuable contributions for clinical applications. In this respect, the study not only presents performance evaluation results but also proposes a strategic framework for future research. Since data from two independent imaging sites were combined without explicit harmonization methods, scanner-related variability may have influenced the learned representations. Although identical preprocessing procedures were applied to both datasets, residual site effects cannot be excluded. Therefore, the reported classification performance should be interpreted with consideration of possible multicenter heterogeneity. Although the overall dataset included 236 subjects, the effective sample size remains relatively modest for deep learning applications involving high-dimensional neuroimaging data. Following quality control, class imbalance and unequal gender distribution may also have influenced model generalizability despite the use of stratified cross-validation. Therefore, to minimize potential bias, the comparative performance of the models should be interpreted primarily based on their mean performance across the cross-validation folds. Although the accuracy achieved (~80%) is lower than that reported by several connectivity-based frameworks, the proposed methodology substantially reduces preprocessing complexity and avoids computationally intensive functional connectivity estimation. Therefore, it may be particularly suitable for rapid screening pipelines or resource-limited clinical environments where computational efficiency is prioritized.

### Limitations and Future Studies

Although the proposed framework demonstrated competitive classification performance while maintaining a relatively simple processing pipeline, several limitations should be acknowledged. First, the study combined two publicly available datasets acquired at different imaging sites without applying explicit harmonization techniques. Although identical preprocessing procedures were applied to all subjects, scanner-related variability and site-specific acquisition differences may have influenced the learned representations. Thus, adopting recently emerging harmonization techniques may reduce variability introduced by differences in measurement methods, thereby improving classification performance. Future studies may validate the proposed framework using larger, more balanced multicenter datasets, along with harmonization methods and leave-one-site-out validation strategies, to better evaluate cross-site generalizability. Second, although the final dataset included 236 participants after quality control, the sample size remains relatively modest for training high-capacity deep learning models using high-dimensional fMRI data. In addition, an imbalance in gender and age distributions across diagnostic groups may have affected model generalizability, despite the use of stratified five-fold cross-validation based on diagnostic labels. Potentially important clinical variables, such as medication status and illness duration, were not incorporated into the analysis. The limited availability of clinical variables across participants precluded their evaluation. As both medication exposure and disease chronicity may influence resting-state brain activity, future studies using more comprehensive clinical datasets may investigate their potential impact on model performance and generalizability. Another limitation concerns the experimental comparison between the 3D and 4D approaches. Although a high-performance computing system was used, not all models and experimental scenarios could be executed at the desired scale simultaneously. Owing to computational resource constraints, the spatiotemporal framework employed the lightweight DarkNet-19 architecture instead of the deeper DarkNet-53 backbone used in the 3D experiments. Consequently, the observed differences between static and spatiotemporal representations may partially reflect differences in network capacity rather than the intrinsic effectiveness of temporal modelling alone. In addition, long training times and high memory requirements were key factors that limited extensive testing of different models and parameter configurations. In particular, the batch size had to be kept lower in 3D and 4D analyses than in 2D approaches reported in the literature, significantly constraining model optimization. Furthermore, the representative raw BOLD analysis was performed using a single (50th) volume selected from each resting-state acquisition. Although this volume was chosen from the temporally stable middle portion of the scan, it may not fully capture temporal variability throughout the entire acquisition. In addition, evaluating multiple representative BOLD volumes or adaptive temporal sampling strategies may provide a more comprehensive characterization of resting-state dynamics. Although this was beyond the scope of the current study, future research may perform systematic comparisons between static and spatiotemporal modelling using backbone architectures with comparable representational capacity, such as 3D VGG and 3D MobileNet. Additionally, comparisons could be made using machine learning models. In addition, incorporating explainable artificial intelligence techniques, such as Grad-CAM or attention-based visualization methods, may improve the biological interpretability of deep learning models and facilitate their use as clinical decision-support tools rather than standalone diagnostic systems.

## 5. Conclusions

This study demonstrates that both data representation and model architecture play critical roles in classifying schizophrenia using resting-state fMRI data, with data representation emerging as the more decisive factor. Among the evaluated approaches, ALFF-based 3D representations provided the highest performance, highlighting the importance of low-frequency BOLD signal characteristics as biologically meaningful and discriminative features. While deep architectures such as ResNet-50 achieved strong, stable results, more computationally efficient models like DarkNet-53 also showed competitive performance. In contrast, incorporating temporal information via 4D hybrid models did not yield significant improvements, suggesting that increased model complexity does not necessarily improve classification accuracy. Overall, the findings suggest that prioritizing biologically relevant data representations, along with balanced model design and computational efficiency, is essential for improving deep learning-based neuroimaging classification systems.

## Data Availability

The datasets used in this study are publicly available. The COBRE dataset was obtained from the Center for Biomedical Research Excellence (COBRE) repository (http://fcon_1000.projects.nitrc.org/indi/retro/cobre.html), and the UCLA dataset (https://openneuro.org/datasets/ds000030/versions/1.0.0) was accessed from publicly available neuroimaging databases. These datasets can be accessed through relevant data-sharing platforms. The analysis scripts and additional materials supporting the findings of this study are available from the corresponding author upon reasonable request.
