# Supplementary material for: Comparative Evaluation of Resting-State fMRI Representations for Schizophrenia Classification Using Deep Learning
Source: Alpha Psychiatry. 2026 Oct 16;27(5):53200. doi: 10.31083/AP53200 (PMC13732001; doi:10.31083/AP53200)
Supplement: Supplementary file 1 [file 2757-8038-27-5-53200-s1.zip › Supplementary Material.pdf]

Supplementary Table 1. Statistical comparison of fold-wise classification performance between different image representations and CNN architectures. Parametric comparisons were performed using paired t-tests, whereas the Wilcoxon signed-rank test was applied when the normality assumption was violated. Effect sizes are reported as Cohen's d (paired t-test) or rank-biserial correlation (Wilcoxon).

Metric	Comparison	Test	Statistic	p-value	Effect size	CI95
Accuracy	ALFF_DarkNet-53 vs 50. Bold_DarkNet-53	paired t	1,742252227	0,156425752	0,779158883	[-3.241 14.161]
Accuracy	ALFF_DarkNet-53 vs Mean_DarkNet-53	paired t	8,718858816	**0,000953135**	3,8991922	[4.616 8.928]
Accuracy	ALFF_DarkNet-53 vs SD_DarkNet-53	paired t	8,28278E-16	1	3,70417E-16	[-9.527 9.527]
Accuracy	ALFF_ResNet-50 vs 50. Bold_ResNet-50	paired t	1,517393571	0,203769705	0,678599035	[-2.478 8.450]
Accuracy	ALFF_ResNet-50 vs Mean_ResNet-50	paired t	1,533887428	0,199839224	0,685975312	[-3.446 11.954]
Accuracy	ALFF_ResNet-50 vs SD_ResNet-50	paired t	2,987082577	**0,040454559**	1,335863939	[0.448 12.260]
Accuracy	50. Bold_DarkNet-53 vs 50. Bold_ResNet-50	paired t	-1,327976224	0,254895442	-0,593889022	[-10.354 3.654]
Accuracy	ALFF_DarkNet-53 vs ALFF_ResNet-50	paired t	-0,355892786	0,73989724	-0,159160092	[-7.710 5.958]
Accuracy	Mean_DarkNet-53 vs Mean_ResNet-50	paired t	-3,118666048	**0,035572455**	-1,394709856	[-6.416 -0.372]
Accuracy	SD_DarkNet-53 vs SD_ResNet-50	paired t	1,372931727	0,241711988	0,613993734	[-5.600 16.556]
AUC	ALFF_DarkNet-53 vs 50. Bold_DarkNet-53	paired t	2,403012919	0,074113795	1,074660047	[-2.062 28.602]
AUC	ALFF_DarkNet-53 vs Mean_DarkNet-53	Wilcoxon	-	0,125	0,6	-
AUC	ALFF_DarkNet-53 vs SD_DarkNet-53	paired t	0,06599096	0,950551632	0,029512054	[-17.661 18.521]
AUC	ALFF_ResNet-50 vs 50. Bold_ResNet-50	paired t	0,462576085	0,667712039	0,206870314	[-13.636 19.088]
AUC	ALFF_ResNet-50 vs Mean_ResNet-50	paired t	0,654677979	0,548410127	0,292780893	[-9.250 14.958]
AUC	ALFF_ResNet-50 vs SD_ResNet-50	paired t	2,19409652	0,093264717	0,981229794	[-4.278 36.514]
AUC	50. Bold_DarkNet-53 vs 50. Bold_ResNet-50	paired t	-1,704753053	0,163442168	-0,762388742	[-25.161 6.017]
AUC	ALFF_DarkNet-53 vs ALFF_ResNet-50	paired t	0,241429822	0,821093343	0,107970699	[-10.206 12.150]
AUC	Mean_DarkNet-53 vs Mean_ResNet-50	paired t	-1,566651668	0,192260077	-0,700627925	[-25.233 7.029]
AUC	SD_DarkNet-53 vs SD_ResNet-50	paired t	2,086525662	0,10524325	0,933122643	[-5.509 38.829]
F1	ALFF_DarkNet-53 vs 50. Bold_DarkNet-53	paired t	1,844117622	0,138933502	0,824714472	[-1.513 7.497]
F1	ALFF_DarkNet-53 vs Mean_DarkNet-53	paired t	3,120921282	**0,035495013**	1,395718428	[0.422 7.218]
F1	ALFF_DarkNet-53 vs SD_DarkNet-53	paired t	-0,217344363	0,838576308	-0,097199354	[-6.419 5.487]
F1	ALFF_ResNet-50 vs 50. Bold_ResNet-50	paired t	1,29734123	0,264279102	0,580188636	[-1.669 4.597]
F1	ALFF_ResNet-50 vs Mean_ResNet-50	paired t	2,468177307	0,06908181	1,103802448	[-0.432 7.344]
F1	ALFF_ResNet-50 vs SD_ResNet-50	paired t	2,73363478	0,052246878	1,222518639	[-0.045 5.749]
F1	50. Bold_DarkNet-53 vs 50. Bold_ResNet-50	Wilcoxon	-	0,0625	-1	-
F1	ALFF_DarkNet-53 vs ALFF_ResNet-50	paired t	-0,280228663	0,793197495	-0,125322068	[-3.796 3.100]
F1	Mean_DarkNet-53 vs Mean_ResNet-50	paired t	-0,777339827	0,480368425	-0,347636939	[-3.255 1.831]
F1	SD_DarkNet-53 vs SD_ResNet-50	paired t	1,496499892	0,208861995	0,669255097	[-2.540 8.480]

Abbreviations: CI95, 95% confidence interval; $p < 0.05$ indicates statistical significance. Cohen's d values of approximately 0.2, 0.5, and 0.8 indicate small, medium, and large effect sizes, respectively.
